# Analysis of the impact of different service levels on the workload of an ambulance service provider

**DOI:** 10.1186/s12913-016-1727-5

**Published:** 2016-09-13

**Authors:** Marco Oberscheider, Patrick Hirsch

**Affiliations:** Institute of Production and Logistics, University of Natural Resources and Life Sciences, Feistmantelstraße 4, Vienna, 1180 Vienna Austria

**Keywords:** Ambulance service, Patient transport, Resource management, Service levels, Dial-a-ride problem, Matheuristic

## Abstract

**Background:**

Efficient transport of non-emergency patients is crucial for ambulance service providers to cope with increased demand resulting from aging Western societies. This paper deals with the optimization of the patient transport operations of the Red Cross of Lower Austria, which is the main provider in this state. Different quality levels of the provided service - expressed by time windows, feasible maximum ride times and exclusive transports - are tested and analyzed on real-life instances to show daily impacts on the provider’s resources. Comparisons of the developed solution approach to the recorded manual schedule prove its advantages. In contrast to previous work in this field, non-static service times that depend on the combination of patients, their transport mode, the vehicle type as well as the pickup or delivery locations are used. These service times are based on statistical analyses that have been performed on an anonymized dataset with more than 600,000 requests.

**Methods:**

To solve the given problem, a matheuristic solution approach was developed that deals with the exact optimization of combinations of requests as a first stage. Subsequently, the identified combinations are used as an input into a Tabu Search strategy, where the vehicle routing is optimized. Three representative days of the year 2012 were chosen for the four regions of Lower Austria to test five different service levels and the quality of the solution method.

**Results:**

For the standard scenario, the operation time of the manual schedule is reduced in the range from 14.1 % to 19.8 % for all tested instances. Even in the best service scenario, the matheuristic computes better results than the manual schedule. The service level has a high impact on the operation time of providers. The relative savings that are achieved by the algorithm are significantly lowered by introducing higher quality standards. The main reason is that less feasible combinations of patients can be generated. This leads to diminished opportunities for patients to be transported at the same time. The results indicate that the implementation of the developed matheuristic in daily planning decisions could decrease operation times significantly.

**Conclusions:**

Managers have to define minimum standards for the punctuality, exclusive transports and excess ride times. This is crucial in order to find a suitable compromise between the service level and an optimized resource management.

## Background

The costs of patient transportation and emergency rescue services have increased steadily over the past 23 years in Austria. They have tripled since 1990 and, due to the predominant demographic trend, there is a high probability of a further increase in future [1]. In order to provide for the needs of aging societies in industrialized countries, efficient planning is crucial for service providers. Besides the cost factor, there is also high pressure on paramedics. These professionals are exposed to and experience a wide range of health problems, e.g., injuries, infectious diseases and post-traumatic stress symptoms [2]. Studies show that this job is intrinsically stressful and that paramedics experience substantially higher levels of musculoskeletal pain [3, 4]. Ambulance routing optimization can help relieve the pressure on paramedics as well as on other resources. Hence, the objective to minimize the sum of operation time and overtime of the paramedics’ deployed shifts for servicing requests was formulated in cooperation with the Red Cross of Lower Austria. Lower Austria is the largest state of Austria with a diverse setting of rural, suburban and urban areas, while, topographically, it covers flat, hilly and alpine areas. The Red Cross is the main provider of these services in Lower Austria, as well as in other states of Austria. In the year 2014, the Red Cross dealt with approximately 2.9 million emergency and non-emergency requests in Austria [5].

Paramedics operate the ambulances to deal with transport requests. Improving scheduling decisions directly affects paramedics and reduces their stress level. Currently, the scheduling is done manually. The dispatchers are aided solely by visualizations of the current positions of vehicles and pending requests. Their dispatching decisions depend on their experience and planning skills, without the support of tools that are able to generate routing and scheduling recommendations automatically. The presented algorithm is a first step towards a decision support system (DSS) that is aimed to help dispatchers improve the quality of routing and scheduling, which additionally leads to a reduction of overtime. Furthermore, maintaining regulated working times and mandatory breaks are important factors to reduce the stress levels of paramedics.

The main aim of the paper is to quantify the implications for the provider if certain minimum standard levels of service are introduced. In contrast to previous work in this field, we incorporate the use of non-static service times that depend on the combination of patients, their transport mode, the vehicle type as well as the pickup or delivery location. Furthermore, a cluster-first route-second matheuristic for the given problem and its decomposition step to compute feasible tasks is introduced. Ex-post analyses with real-life data show the advantages of using the algorithm.

### Outline and literature review

The underlying problem of the provider was introduced in several meetings and later formulated. It shares multiple characteristics with problems that can be found in Operations Research literature and is an extension of a static multi-depot heterogeneous dial-a-ride problem (MD-H-DARP) [6]. Static denotes that complete information about requests and deployable shifts is assumed and no changes to this information occur throughout the day. The extensions made to the problem defined by [6] concern mandatory breaks, as also considered in [7–9], as well as the return policy of the provider and varying service times at pickup or delivery locations that depend on the transport mode, the vehicle type, the combination of patients and the pickup or delivery locations, e.g., nursing homes, hospitals or wards. The service times are derived from statistical analyses. Varying service times are considered by [9], however, they solely depend on the transport mode, which can be ambulatory or wheelchair. The return policy of the provider is also not commonly used for these types of problems. A vehicle must return to its predefined depot if there is no direct consecutive request. This policy is implemented due to the fact that the main part of the fleet is also deployed for emergency rescue services. To conclude, our approach extends the MD-H-DARP by using mandatory breaks, having a different return policy and introducing non-static service times.

Due to the extensive number of publications on the DARP, only those that share similar features with the given problem are considered and mentioned in this paper. For further information on the DARP as well as pickup and delivery problems, refer to [10–12]. Most publications contain problems with single depots, e.g., [9, 13, 14], however, in the recent years, some consider multiple depots, e.g., [6, 7, 15, 16]. Driver related constraints concerning maximum route durations and mandatory breaks are included in [7–9, 17]. Homogeneous users were the point of focus in most publications, e.g., [13, 18, 19], however, there are several authors that deal with heterogeneous cases, e.g., [6, 7, 9, 20, 21]. Solution approaches developed by the authors of these publications are as numerous as the extensions of the DARP and contain heuristics, metaheuristics, matheuristics and exact algorithms.

To solve the given problem of the Red Cross of Lower Austria, a matheuristic was implemented. In accordance with [22], it can be classified as a decomposition approach using a cluster-first route-second strategy. The first step is the enumeration of all combinations of patient transports that observe the given constraints. These feasible combinations are called tasks and are identified by a recursive depth-first search (RDFS). Subsequently, they are combined by solving a set partitioning problem with the objective of minimizing service time and driving time with patients on board, i.e., transport time. This first stage of the proposed algorithm is computed by a similar approach as used by [7] to transport handicapped people in Berlin. Next, these tasks are heuristically assigned to shifts to generate an initial solution for a Tabu Search metaheuristic.

The algorithm is tested with real-life data from 2012. Different scenarios are defined to solve daily instances with up to 848 requests and 178 disposable shifts. The results of the algorithm are compared to the manual schedule in ex-post analyses for various service levels. A survey on the quality of service in dial-a-ride operations is given in [23]. The authors highlight that service quality is defined in literature as technical quality or customer-based quality. In [24], it is specified that “technical quality refers to the conformity to specifications used by the provider of the service to set a level of quality". This type of quality is typically measurable, as it is based on objective criteria, while customer-based quality is considered to be more subjective. In this paper, service levels are expressed by varying time windows, the proportion of exclusive transports as well as varying feasible maximum ride times and handled as constraints to generate a single solution. In [9] a different approach was followed by incorporating similar quality measures in a multicriteria algorithm to create a set of non-dominated solutions, i.e. a Pareto front. In contrast, we define minimum standards through a variation of the service measures to reveal the trade-off of an increased service level for the patient and the pressure on the required resources of the provider. Comparisons of the used approach to the recorded manual schedule show the advantages of an optimized schedule.

### Problem description

For the extended static MD-H-DARP, two types of requests are given, namely patient transports and transport of goods. The transport of goods includes organs, samples, blood conserves and materials. These subcategories are not further distinguished concerning their requirements, as transports of goods rarely occur. Patient transports differ in terms of their transport mode. Ambulant patients and patients with their own wheelchair are transported in patient seats. In the latter case, the wheelchair is stored inside the vehicle and the patient is transferred to the wheelchair upon arrival. Recumbent patients have to be transported on a stretcher. Lastly, patients can also be transported on a carrying chair.

A heterogeneous fleet of vehicles located at several depots is available to service the given requests. There are two types of vehicles that differ by the number of paramedics on board, as well as the number and transport modes of patients. An auxiliary ambulance (AAM), which is similar to an estate car, is operated by a shift consisting of one paramedic. It has a maximum capacity of three ambulant patients. A patient transport ambulance (PTA) is specially equipped to handle the needs of a maximum of two patients. These patients can be transported in a carrying chair, on a stretcher or in a patient seat. For the PTA, any combination of two patients is permitted except the one with two patients in need of a stretcher. The shift of a PTA is composed of two paramedics who perform the boarding and deboarding of patients. Goods can be transported with both types of vehicles.

The transport modes differ in terms of their feasible combinations as well as their service times needed at the pickup and delivery locations. This means that different service times are used for various nursing homes or hospitals and even different wards of a hospital. These service times are based on statistical analyses of more than 600,000 requests. Due to greater efficiency, changes to service times occur if two or three patients get picked up from or are delivered to the same location. These changes depend on the transport modes and whether the patients are served at the same ward of a hospital.

Most requests can be combined with other requests, while certain ones have to be executed individually. Exclusive transports are more convenient for patients, however, there are also medical reasons, e.g., patients with radiation therapies or mental health problems. The assignment of goods requests is done individually for both types of vehicles, indicating that patients cannot be transported at the same time as blood conserves, materials, samples or organs. Combining patient transports may result in detours for the patients on board. A requests’ maximum exceedance of the direct ride time is depending on the computed shortest path from the pickup to the delivery location.

In summary, each request is specified by a transport mode, a pickup and delivery location, a time window at the pickup location, a maximum ride time and service times at both locations as well as if it is an exclusive transport.

Vehicles start at a depot at the beginning of a shift of the assigned paramedic(s). They have to return to the depot at the end of the shift, however, the return can be delayed by a maximum, predefined overtime allowance. According to the return policy of the provider, vehicles also have to return to the depot if idle. To comply with Austrian law and organizational rules, breaks of 30 minutes have to be taken between the beginning of the third and the end of the sixth hour of a shift. Breaks can be taken at depots or at one of the pickup or delivery locations. The number and type of vehicles used, as well as the start and end of a shift on a given day, is derived from the manual schedule used by the Red Cross.

The objective (1) is to minimize the total operation time of the deployed shifts and to penalize overtime.

1 $$\begin{array}{*{20}l} \min &\sum_{s \in S} (t_{s} + s_{s} + d_{s} + w_{s} + \gamma o_{s})  \end{array} $$

The operation time of a shift *s*∈*S* includes transport times *t*_*s*_, service times *s*_*s*_ for boarding and deboarding of patients, drive times with an empty vehicle *d*_*s*_ as well as wait times *w*_*s*_. Overtime *o*_*s*_ are feasible as long as they are lower than a predefined maximum, but they are not desirable. Thus, they are penalized by *γ* and integrated into the objective. Wait times may occur between the service of two requests, while waiting is not permitted with patients on board. As shifts, in general, must return to their depot if idle, the following convention is used for waiting: if the start of the next request of a shift does not leave enough time to return to the depot and drive to the next pickup location, the shift is allowed to wait at its current position. After the wait time, the vehicle is driven directly to the next pickup location.

In summary, the underlying problem has the following constraints:

- Each request has to be served.
- The capacities of the vehicles have to be respected:
  - An AAM can transport up to three ambulant patients.
  - A PTA has a maximum capacity of two patients, with a maximum of one recumbent patient on a stretcher.
- The time windows at pickup locations have to be met.
- Requests can be exclusive, meaning they cannot be combined with other requests.
- Maximum ride times cannot be exceeded.
- The paramedics have given shifts and mandatory breaks.
- Paramedics must return to their depots if idle.

## Methods

The following section introduces the solution approach, gives an overview of the applied statistical analyses and describes the test setting of the numerical studies.

### Matheuristic approach

**Fig. 1:**
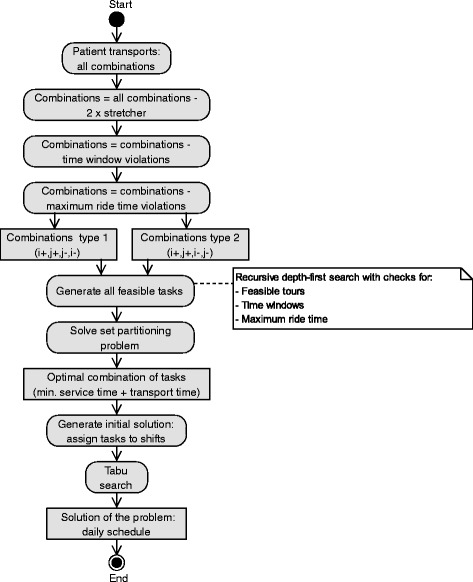
Activity diagram of the implemented algorithm. An overview of the sequence of activities from building the tasks to optimizing the schedule
- the combination would lead to a time window violation or
- the combination violates the maximum ride time of at least one of the patients.

To solve the given problem, a matheuristic solution approach was introduced. Figure 1 shows the implemented algorithm. As a starting point, all combinations of two patient transports are generated. Next, combinations are eliminated if

**Fig. 2:**
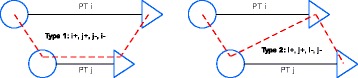
Possible combinations of two patients. Type 1 (*left*) and Type 2 (*right*) are generated to get all feasible successors and predecessors of a request

- both patients require a stretcher,

The remaining combinations can be categorized as shown in Fig. 2. In both categories, patient *i* is picked up before patient *j*. In the first category, patient *j* is delivered before patient *i*, while in the second category, patient *i* is delivered before patient *j*. After computing the feasible combinations of two patients and, thereby, generating all direct predecessors and successors of a patient transport, an RDFS is executed for PTAs and AAMs separately. The RDFS for AAMs solely uses combinations of ambulant patients as input. In the two RDFSs, all feasible tasks are created that comply with time windows, capacities, maximum ride times and a maximum duration of 330 minutes. This maximum duration is set to make sure that breaks can be taken at feasible times. Tasks are variably long combinations of patient transports, where, at any point in time, a minimum of one patient and a maximum of two patients in a PTA and three patients in an AAM are present.

In the next step, all tasks are input into the software “Fico Xpress 7.7" to solve a set partitioning problem. The solution indicates the optimal combination of tasks containing all patient transports, while having an exact minimum of transport plus service times. This optimal combination is returned to the algorithm. Subsequently, all exclusive transports are added.

By the use of a cheapest insertion heuristic that includes a rejected-reinsertion operator [25], these tasks are assigned to shifts to generate an initial solution for a metaheuristic based on Unified Tabu Search [26]. This initial solution, as well as intermediate solutions in the Tabu Search, are allowed to be infeasible due to violations of time windows and maximum overtime regulations.

The objective of the metaheuristic equals Eq. (1) reduced by the transport times *t*_*s*_, since these are predetermined by the generated tasks. The Tabu Search applies the neighborhood operators, string relocation, string exchange and 2-opt*, in the given order. If a solution cannot be improved by the use of the current operator, the subsequent operator is applied in the next iteration. After the use of an operator a tabu status is set to the operation’s attributes to avoid their reversal for the next *θ*=(*log*(|*shifts*|∗|*tasks*|))^2^ iterations. The tabu duration *θ* is dependent on the size of the problem and was obtained after a number of parametrization approaches. The tabu status of an operation can be overruled by an aspiration criterion if the new solution has a lower objective value than the best known solution having that attribute. After a predefined run-time of the metaheuristic, the algorithm stops and the best found solution is returned. For the included relaxation scheme to explore infeasible solutions, self-adjusting positive parameters *α* (overtime) and *β* (time windows) are introduced. Similar to [10] they are used in a cost function to facilitate the exploration of the solution space. In each iteration these parameters are modified by a factor 1+*δ*. If a solution is feasible in terms of time windows, *β* is divided by 1+*δ*, while it is multiplied by 1+*δ* otherwise. The same rule applies to *α*. To diversify the search, a penalty factor is added to the cost function in the same way as in [18]. Hence, for controlling the diversification the parameter *λ* was introduced. The parameters of the metaheuristic were tested for the standard scenario on an average-sized instance. Therefore, the smallest instance of Region 3 was tested with 36 test runs in order to find the best combination of *λ*∈{0.010,0.015,0.020,0.025} and *δ*∈{0.1,0.2,0.3,0.4,0.5,0.6,0.7,0.8,0.9}. After 10^5^ iterations the parameter setting of *δ*=0.6 and *λ*=0.010 resulted in the best objective value and was used for the numerical studies. During the computation, the forward time slack [27] of tours is computed to reduce wait times.

### Statistics of service times

The statistics are based on 612,453 requests that were performed by the Red Cross of Lower Austria in 2012. Paramedics operate a device that is installed inside of the vehicle to report their current status. Service times at pickup and delivery locations are collected by time stamps of various status updates.

The input data does not follow a normal distribution, thus the median is used as input for the matheuristic. In total, six different categories that follow the transport modes were used for the calculation of service times. Namely, they include goods, patient-owned wheelchairs, ambulant AAMs, ambulant PTAs, recumbent stretchers and carrying chairs.

Medians were computed for all locations separately if a hospital or nursing home serves as pickup or delivery location for more than 500 times. For hospitals that were the origin of more than 1,000 pickups or deliveries, wards were used as units of distinction if they had more than 100 data entries. Table 1 shows the number of locations or wards that have been considered.

**Table 1:** Number of units of distinction for median computation [#]

Type	Locations with ≥	Locations with ≥	Wards with ≥
	500 requests	1,000 requests	100 requests
Pickup	35	26	190
Delivery	42	29	201

To compute the changes of service time due to a combination of patients, the following rules for AAMs and PTAs have been defined together with the provider. These are applied if more than one patient is served at the same location.

The first step is to compute the maximum of the single service times of the patients. Subsequently, the following cases are distinguished:

- Same ward:
  - If at least one patient is ambulant, the maximum single service time is increased by 20 % per additional patient. The addition of 20 % is caused by an increasing administrative effort for the other patient(s) (AAM/PTA).
  - For all other combinations, i.e., none of the patients is ambulant, 70 % of the maximum single service time is added (PTA).
- Different ward:
  - If the transport modes consist of a combination of ambulant patients and patients with their own wheelchair, a parallel pickup or delivery is possible by the two paramedics. Thus, only the maximum of the single service times is used (PTA).
  - For all other combinations of transport modes, a sequential order of the movements of patients is assumed (AAM/PTA).

### Numerical studies

For the numerical studies, three days in 2012 were chosen. These represent the day with the highest, the lowest and a median number of requests, whereas weekends and holidays were excluded as statistical outliers. The state of Lower Austria is geographically divided into four regions and this clustering is also used in the numerical studies. In total, this leads to twelve real-life instances containing the input data of three days per region. Data of effectively employed shifts by the provider were not available. Therefore, the following procedure to estimate this data was derived with input from the provider: Shifts of eight hours were generated, with start times dependent on the first service request of a vehicle on a certain day. If the same vehicle appears later on the same day and after the end of the first shift in the data, a new eight hour shift was introduced. The maximum overtime of a shift are set to 120 minutes. Overtime exceeding this value lead to infeasible solutions, while overtime below or equal to this value are penalized by the factor *γ*=0.5 per minute. This factor was chosen since overtime result in a surcharge of 50 % for the provider.

In total, five different service levels (scenarios) were tested on the twelve real-life instances (Table 2). The service levels equal minimum standards that the provider guarantees all clients. It is a managerial decision that defines the worst case of detours, a share of exclusive transports and pickup time window spreads. The standard scenario is the current aim of the Red Cross of Lower Austria. It has time windows of 30 minutes, excess ride times of 100 % and 10 % exclusive transports. Starting from this scenario, different service levels are defined by widening or narrowing time windows, increasing or decreasing the share of exclusive transports and allowing shorter or longer maximum ride times. Excess ride times are added to direct ride times to calculate the maximum ride times of patients. To compute an excess ride time, the direct ride time is multiplied by a varying percentage according to the given scenario. Additionally, a lower bound of 10 minutes and an upper bound of 30 minutes is used for the evaluation of excess ride times.

**Table 2:** Parameters of the five tested scenarios

Scenario	TW length [min.]	Excl. transports [%]	Excess ride time [%]
Excellent	20	20	60
Enhanced	25	15	80
Standard	30	10	100
Reduced	35	5	120
Bad	40	0	140

All tests were performed on a single workstation with an Intel Core i7-3930K with 3.2 GHz and 64 GB RAM, with MS-Windows 7 as the operating system. The Tabu Search was implemented in C++ and executed with a run-time of 10 minutes. This was defined as an acceptable wait time for a user. Tests further showed that improvements in solution quality diminish after this run-time.

## Results

The results section is organized as follows: it starts with an overview of parameters of the input data, gives an overview on tested indicators for all instances and ends with a detailed analysis of a representative day.

### Results of the data analysis

Table 3 shows the input of the twelve test instances in terms of number of requests and deployable shifts on the given days in a certain region.

**Table 3:** Number of requests and deployable shifts per region and day [#]

	Minimum		Median		Maximum	
Region	Requests	Shifts	Requests	Shifts	Requests	Shifts
1	304	96	382	94	391	100
2	372	106	461	112	515	113
3	551	165	600	167	780	184
4	558	138	789	167	848	178

In Table 4, the distribution of the transport modes is given for the four regions. In total, 60.6 % of the patients require a carrying chair, 26.3 % are ambulant patients and 9.4 % need a stretcher. A transport of a patient with his or her own wheelchair is relatively seldom and the transportation of goods is quite uncommon.

**Table 4:** Number of requests per region and distribution over the different transportation modes

Region	Requests	Ambulant	Carrying	Own	Recumbent	Goods
			chair	wheelchair		
	[#]	[%]	[%]	[%]	[%]	[%]
1	1,077	31.3	56.7	2.0	9.8	0.1
2	1,348	25.4	60.2	2.1	10.9	1.3
3	1,931	30.2	58.8	1.9	8.6	0.4
4	2,195	21.0	64.4	5.6	8.9	0.1
Total	6,551	26.3	60.6	3.2	9.4	0.4

Table 5 shows the number of depots in the different regions as well as the total number of shifts over the three scenario days and the proportion of PTAs to AAMs. Of note is that Region 4 only employs 10 % of AAMs, while Region 1 and 3 employ approximately 30 % of AAMs and have a significantly higher share of ambulant patients.

**Table 5:** Number of depots, deployable shifts and proportion of PTAs to AAMs in the different regions

Region	Depots [#]	Shifts [#]	PTAs [%]	AAMs [%]
1	19	290	69.3	30.7
2	25	331	78.2	21.8
3	45	516	70.0	30.0
4	45	483	89.6	10.4
Total	134	1,620	77.4	22.6

### Results of all tested instances

Table 6 shows the objective values of all tested scenarios. The objective value is expressed in minutes and is the sum of operation times (transport/service/wait time and driving empty) and overtime * 0.5 of all shifts.

**Table 6:** Objective values of all 60 tested instances [min.]

Region	Day	Manual	Excellent	Enhanced	Standard	Reduced	Bad
1	Min	20,848	18,136	17,292.5	16,459.5	15,943	15,508
	Med	23,870	20,715	19,888.5	19,252	18,812.5	18,156
	Max	23,854	21,579	20,785	19,744.5	18,962.5	18,297
2	Min	24,053.5	20,110.5	19,303	18,628	18,257.5	17,743.5
	Med	29,747.5	25,929.5	24,565.5	23,430	22,681.5	21,977.5
	Max	30,801	27,879.5	25,943.5	24,916	24,377.5	23,705
3	Min	35,896	30,191	29,231	27,928	26,977.5	26,337.5
	Med	37,581.5	32,697.5	31,263	30,169.5	29,140	28,045
	Max	46,225	39,251.5	37,592.5	36,407.5	35,543.5	34,745.5
4	Min	29,870.5	25,535.5	24,548	23,884.5	23,303	22,863
	Med	39,243.5	34,751	33,057	31,782	31,020	30,296
	Max	45,384	38,996	37,197	36,346	35,076	34,508

**Fig. 3:**
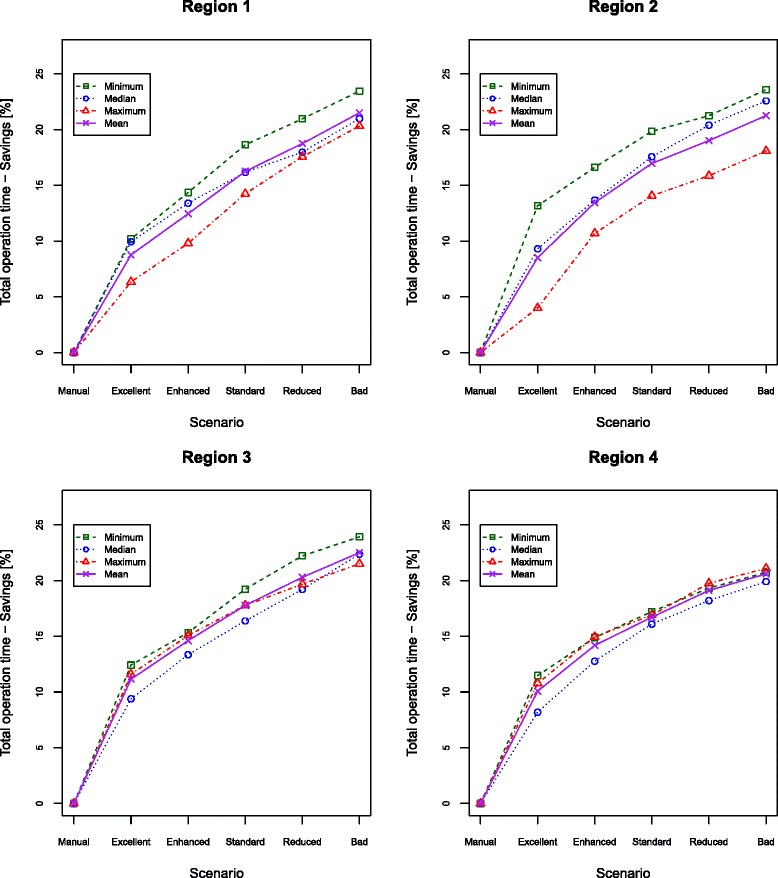
Relative savings of operation time. The relative savings of operation time in relation to the manual schedule for the different scenarios of all tested instances

For comparisons between different scenarios, the relative savings of operation time are more meaningful than the objective values. Figure 3 shows these savings with the operation time of the manual schedule used as reference.

The savings are highly dependent on the minimum standard ensured by the provider and differ from instance to instance. For all instances, the savings of operation time increase with decreasing service level. The mean of the savings over all tested instances is 16.3 % ranging from 4.0 % (Region 2/Max/Excellent) to 23.9 % (Region 3/Min/Bad). Savings in Region 3 and 4 follow a comparatively narrow range, while those of Region 1 and 2 show a considerable gap between the minimum and the maximum day. In particular, the reduction of operation time in Region 2 depends on the day, where a maximum difference of 9.2 % is present between the minimum and maximum day of the excellent scenario.

Table 7 shows the number of chosen tasks after solving the set partitioning problem. For most of the days, the number of requests that have been combined to tasks in the manual schedule is relatively close to the excellent service scenario. The main difference can be seen for the maximum day of Region 2, where the excellent scenario uses 41 tasks more than the manual schedule. This setting also shows the lowest savings of all performed tests.

**Table 7:** Number of requests that have been combined to tasks per test instance [#]

Region	Day	Requests	Manual	Excellent	Enhanced	Standard	Reduced	Bad
1	Min	304	242	246	230	206	198	185
	Med	382	311	311	292	272	251	239
	Max	391	311	319	298	273	256	239
2	Min	372	305	303	275	252	244	228
	Med	461	365	376	348	322	297	279
	Max	515	382	423	384	351	336	315
3	Min	551	463	449	410	376	353	328
	Med	600	493	485	446	404	377	351
	Max	780	609	604	562	520	488	446
4	Min	558	467	452	421	389	370	343
	Med	789	634	634	583	542	492	461
	Max	848	669	666	618	567	522	498

Other indicators of note are the total number of shifts that execute tasks and the peak of shifts operating in parallel (Table 8). The number of shifts shows the same overall trend as the number of tasks, which decrease with lower service levels, however, this is not valid for all tested instances.

**Table 8:** Total number and peak of shifts that are deployed in parallel per test instance [#]

Region	Day	Manual	Excellent	Enhanced	Standard	Reduced	Bad
1	Min	96/47	77/36	71/35	70/34	67/32	69/35
	Med	94/49	78/46	77/40	74/40	75/39	72/37
	Max	100/45	84/42	79/42	76/39	75/37	70/37
2	Min	106/42	82/37	80/37	72/33	71/34	75/33
	Med	112/56	89/55	90/49	87/47	88/46	84/47
	Max	113/51	96/54	89/50	90/48	88/46	90/48
3	Min	165/68	126/62	117/59	114/54	110/51	108/51
	Med	167/79	132/67	120/64	118/59	113/60	104/55
	Max	184/91	144/79	143/74	139/73	134/72	130/74
4	Min	138/55	113/48	108/47	106/44	99/45	100/44
	Med	167/79	136/75	132/74	126/70	128/65	124/66
	Max	178/87	140/92	142/83	139/79	136/76	139/74
Total		1,620/749	1,297/693	1,248/654	1,211/620	1,184/603	1,165/601

For all tested instances, the number of deployed shifts is greatly reduced. On average, the bad service scenario requires approximately 10 % less shifts than the excellent service scenario. The highest reduction between these two scenarios can be found for the medium day of Region 3 with a difference of 28 deployed shifts (21 %).

The peak of the manual schedule can be directly derived from the data, which appears around noon. With two exceptions (maximum day of Region 2 and Region 4), the peak use of vehicles is lower for the excellent scenario compared to the manual schedule and is generally decreasing with the service level. The values go up to a reduction of 24 shifts (Region 3/Bad) and show a mean reduction of 9.6 shifts.

### Detailed results of a representative day

**Fig. 4:**
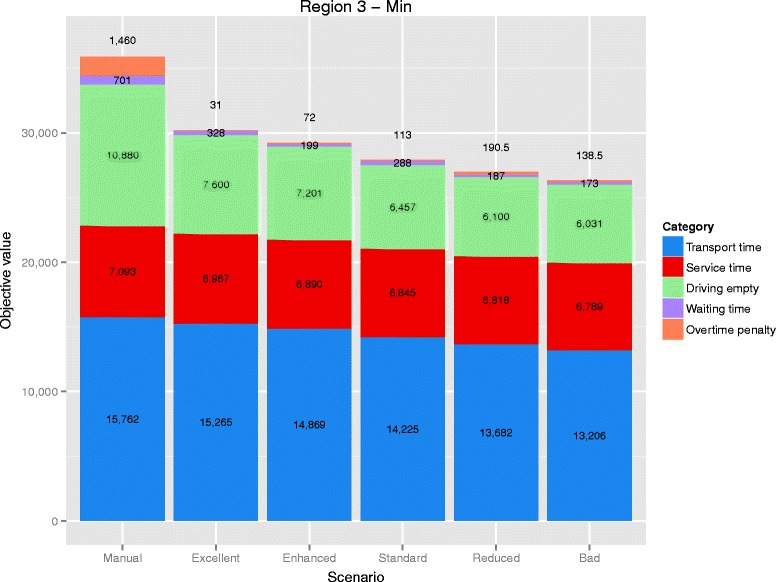
Objective value. Composition of the objective value for the different scenarios of the minimum day of Region 3

To give more detailed insight, the smallest instance of Region 3 was chosen due to its average size in relation to the other instances. In total, there have been 165 shifts at 39 different depots available to serve 551 requests. After 10 minutes run-time of the algorithm, 126 shifts have been deployed in the excellent service scenario, while this number decreases to 108 in the bad service scenario. In the manual schedule, 463 tasks were built out of the given patient transports. The algorithm combined more requests. The number of tasks is decreasing with service level and ranges from 449 in the excellent service scenario to 328 in the bad service scenario. Table 9 summarizes the detailed results, which are illustrated in Fig. 4. The column “Overtime" contains the sum of overtime of all deployed shifts, whereas each deployed shift has to comply with the maximum overtime of 120 minutes. The total overtime of the manual schedule are also significantly reduced for other instances in all tested scenarios. In Table 9, overtime are given in minutes, while in Fig. 4 objective values are displayed and, therefore, the overtime are multiplied by 0.5. The proportion of the different components of the objective function is representative for all tested instances. Service time and transport time account for approximately 75 % in the algorithmic results and around 64 % in the manual schedule. This also indicates the potential of the algorithm as it solves these two components of the objective to an exact minimum.

**Table 9:** Indicators of the five tested scenarios for Region 3/Min

Scenario	Shifts	Tasks	Transport	Service	Driving	Wait	Overtime
	[#]	[#]	time	time	empty	time	[min.]
			[min.]	[min.]	[min.]	[min.]	
Manual	165	463	15,762	7,093	10,880	701	2,920
Excellent	126	449	15,265	6,967	7,600	328	62
Enhanced	117	410	14,869	6,890	7,201	199	144
Standard	114	376	14,225	6,845	6,457	288	226
Reduced	110	353	13,682	6,818	6,100	187	381
Bad	108	328	13,206	6,789	6,031	173	277

Savings of operation time of the algorithm account for 12.4 % in the excellent service scenario and are increasing with decreasing service quality to 23.9 % in the bad service scenario.

**Fig. 5:**
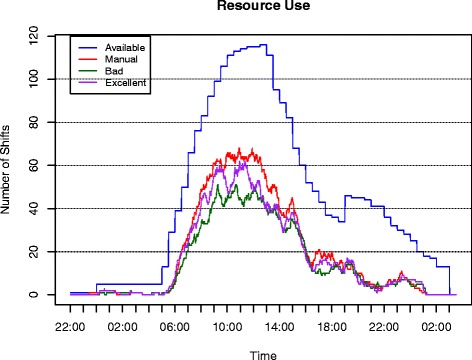
Resource management. Available and used resources for the manual schedule, the bad and the excellent service scenario

Figure 5 shows the deployed and available shifts over the course of the day. In order to improve readability, only the curves of the manual schedule, the excellent and the bad service scenario are displayed. Peaks are reached around noon, whereas the number of requested services increases steadily around 6 a.m. and starts to fall around 1 p.m. The time between 6 a.m. and 5 p.m., i.e., the area of a high number of requested services, is also the time interval during which it is possible to combine requests to tasks most efficiently. Hence, the main difference between the curves can be found at peak times. The peak of the deployed shifts of the manual schedule shows a value of 68, while it is reduced to 62 in the excellent service scenario and to notably 51 for the bad service scenario.

## Discussion

The results show the potential benefits of the proposed algorithm and a computer aided DSS for routing and scheduling. The general aim of the provider is expressed by the standard scenario. In this scenario the operation time of the manual schedule is reduced in the range from 14.1 % to 19.8 % for all twelve instances tested. Large savings could be obtained in all tested regions. Hence, this is achievable despite differences between regions in terms of the geography, the patients mobility and the fleet composition.

The computed savings are highly dependent on the applied minimum standard of the provider. Narrow time windows and small exceedances of direct ride times lower the potential number of combined requests and, therefore, lead to a high amount of individual transports. Combinations of patients lead to a reduction of the number of vehicles that have to be operated in parallel, thus relieving resources. As the provider also uses PTAs in cases of emergencies, a lower quality standard increases the reliability of having short response times for this time-critical service.

The dependence on the scenario is also described by [10], who state that DARPs often contain conflicting objectives of minimizing operational costs, expressed by operation time and maximizing quality. This negative correlation between productivity and quality in dial-a-ride services is also mentioned by [23]. As problems and objectives in the literature differ from this study and comparisons to manual schedules are rare, findings of other studies can only be used to a limited extent to investigate the potentials of DSS for routing and scheduling. For example, [28] solves a DARP in a mid size US city and presents a comparison of the used solution approach to a manual schedule. A medium sized instance (357 requests/36 vehicles) is improved by 22 % and a large instance (680 requests/48 vehicles) by 12 %. By varying time windows and maximum ride times, the medium sized instance was used to compare different service scenarios. The impact on the minimization of total miles driven, which was their objective function, shows a similar trend as the variation of operation times in this paper.

The main reason for the increase in operation time with higher service levels is the number of tasks that are built in the first stage of the algorithm. The lower the service quality, the more requests can be combined to tasks. For example, only 2,777 feasible tasks are built for the largest instance with 848 requests under excellent service conditions. Under bad service conditions, 5,918,487 feasible tasks are used as input to the set partitioning problem for the same instance. Hence, the algorithm has a larger set to choose from, when the service level decreases. The algorithm picks the set of tasks with the minimum sum of transport and service time. This represents the main share of the objective value. The optimization of operation times also corresponds to a lower number of deployed shifts. The exceptions of this trend can be explained by the fact that minimizing the number of deployed shifts is not part of the objective function.

In [28], a similar approach was used to build tasks, which are referred to as mini-routes. It is stated that combining requests to mini-routes is the main advantage of an algorithm compared to a manual schedule, due to the complexity of this activity. A similar conclusion is drawn in [7], where heuristic clustering of requests was compared to an optimal clustering approach. The findings of the authors are that optimal clustering reduces the number of requests about 10 % more than doing so heuristically. A comparison to a manual combination of requests is not given, however, it is valid to assume that the heuristic used is at least as good as a manual approach.

## Conclusions

The results show that the service level determines the resource management of the provider. Managers define the minimum standards for punctuality, exclusive transports and excess ride times. Analyses as done in this paper show the impact on the used resources, i.e., the number of vehicles required to operate in parallel for ambulance services. Providers have to find a suitable compromise between the service level for the patients and resource management. A DSS for routing and scheduling that respects the given minimum standards can be implemented to improve the quality of the dispatchers’ decisions.

In the future, it is planned to continue work on this problem. Adding a reclustering phase to the algorithm could be beneficial due to the broadening of the solution space. By reducing the neighborhood size through clustering, solutions of good quality are obtained quickly. However, after observing a stagnation of the search process, a reclustering phase could be started to explore new areas of the search space. The main focus will be on the transformation of the static model into a dynamic one. Such a model is the next step to creating and implementing a DSS for the routing and scheduling of ambulance services. To assess the quality of a dynamic approach, a comparison to the solutions of the static algorithm will be of interest. The focus of such work will be to see to what extent not knowing all information in advance will contribute to increasing operation times.

## Abbreviations

AAM: Auxiliary ambulance
DARP: Dial-a-ride problem
DSS: Decision support system
MD-H-DARP: Multi depot heterogeneous dial-a-ride problem
PTA: Patient transport ambulance
RDFS: Recursive depth first search
TW: Time window
